# Stakeholder preferences on digital health in Germany: a health preference study protocol

**DOI:** 10.1186/s12913-026-14216-8

**Published:** 2026-04-29

**Authors:** Axel Mühlbacher, Andrew Sadler, Ann-Kathrin Fischer

**Affiliations:** 1 Health Economics and Health Care Management, Hochschule Neubrandenburg, Brodaer Straße 2, 17033 Neubrandenburg, Germany; 2 Gesellschaft für empirische Beratung mbH, An-Institut Hochschule Neubrandenburg, Berlin, Germany

**Keywords:** Health preference research, Discrete choice experiment, Best worst scaling, Study protocol, Digital health interventions

## Abstract

**Background:**

Digital health interventions (DHIs) are increasingly shaping healthcare systems. However, their adoption is often hindered by fragmented evaluation frameworks that fail to reflect clinical, technical, economic, and societal value dimensions. This study protocol outlines the design of a stakeholder-informed preference study aimed at developing a multidimensional evaluation framework for DHIs in Germany.

**Methods:**

The study applies a prospective health preference research design combining discrete choice experiments (DCEs) and best-worst scaling (BWS) Type 1. Based on a systematic review, qualitative interviews (*N* = 10), and expert consultations, four decision models were developed reflecting the value dimensions of DHIs at the subject, interaction, system, and societal levels. A total of 31 descriptive and two continuous attributes (out-of-pocket costs and daily time investment) were identified and structured into a partial profile design. These models form the basis for four DCE modules. A total of 4,250 to 4,300 participants from the general population and healthcare provider network will be recruited and randomly assigned to one DCE module. Each participant will complete nine choice tasks, each followed by a question to approximate real-world acceptance. Following the DCE module, a BWS Type 1 task will be administered to rank all 31 attributes. A joint likelihood model will be used to combine DCE and BWS data using shared attributes as anchors. The final survey instrument was pretested with six individuals to assess usability, clarity, and response behavior under realistic conditions. Planned analyses include conditional logit, mixed logit, and latent class models. Background variables including self-reported health status, experience with digital health, and key sociodemographic characteristics will be used for subgroup analyses. Quality assurance measures include pre-launch testing of the survey infrastructure and automated detection of inattentive or non-human responses using indicators such as completion time anomalies, repetitive patterns, and semantic plausibility of open-ended input.

**Discussion:**

This study will generate robust evidence on stakeholder preferences for DHIs. It will quantify attribute importance, willingness to pay, willingness to invest time, and subgroup-specific heterogeneity. The resulting multidimensional framework aims to support the design, implementation, and reimbursement of DHIs in alignment with stakeholder values.

**Trial registration:**

Not applicable.

**Supplementary Information:**

The online version contains supplementary material available at 10.1186/s12913-026-14216-8.

## Introduction

### The evaluation challenge of digital health interventions

As healthcare systems worldwide undergo rapid digital transformation, digital health interventions (DHIs) have emerged as pivotal tools for enhancing care delivery, improving patient outcomes, and addressing resource constraints [1–3]. These technologies including telehealth services, mobile health apps, wearable devices, and electronic health records are reshaping the way care is accessed, delivered, and experienced [4–6]. DHIs are defined as digitally delivered or supported health services that address the needs of individuals such as patients, clients or caregivers, as well as healthcare providers, health system managers and data services [2]. However, their integration faces critical evaluation challenges that this study seeks to address.

In Germany, the adoption of DHIs is propelled by rising healthcare costs, workforce shortages, and an expanding regulatory framework. Certified digital applications such as DiGAs (Digital Health Applications) [7], DiPAs (Digital Care Applications) [8], and electronic patient records (ePA) [9, 10] exemplify the growing integration of digital solutions across care settings. Legislative initiatives including the eHealth Act (2015), the Digital Supply Act (2019), and the Digital Act (2024) have reinforced this shift [11–13].

Germany’s national digital health strategy, as outlined in the 2024 Digital Act, emphasizes evidence-based integration of DHIs into routine care. Yet, reimbursement and funding decisions remain fragmented and often lack evaluation criteria informed by real-world stakeholder perspectives. This underscores the need for robust, multidimensional assessment frameworks that reflect user and provider priorities while accounting for clinical, economic, and societal value.

Current evaluation approaches tend to be fragmented, focusing narrowly on clinical effectiveness or technical feasibility, without adequately addressing system-wide integration, user satisfaction, or broader societal impact. This limitation hampers stakeholders’ ability to make informed decisions about the adoption, implementation, and funding of DHIs. For providers, this adds complexity to clinical integration; for patients, it creates uncertainty regarding safety, trust, and usability; and for developers and payers, it generates a volatile market environment marked by unclear value propositions and regulatory ambiguity. Existing models often neglect patient and provider perspectives, limiting their applicability to real-world decision-making.

In response to these gaps, health preference research (HPR) provides a structured methodology to quantify stakeholder priorities and trade-offs. By integrating Discrete Choice Experiment (DCE) and Best Worst Scaling (BWS) methods, this study aims to develop a comprehensive, stakeholder-informed framework for evaluating DHIs in the German healthcare context. To date, few studies have addressed this need by capturing both user and provider preferences across all relevant dimensions of digital health value.

### Research question

This study seeks to develop a stakeholder-informed evaluation framework that systematically assesses the acceptance, integration, and impact of DHIs across clinical, technological, economic, and sociocultural domains.

Specifically, the study investigates the following guiding questions:

- Which outcomes and metrics do patients and providers consider most relevant for evaluating the clinical effectiveness and user experience of DHIs?
- How do different stakeholder groups perceive the value and challenges associated with integrating DHIs into healthcare delivery?
- What factors drive or hinder the acceptance and adoption of DHIs across diverse patient populations and clinical settings?

## Methods and study design

### Study objectives

This study aims to develop a stakeholder-informed evaluation framework that captures the clinical, technological, economic, and sociocultural value dimensions of DHIs. Using HPR approach in accordance with established best practices in health economics and health technology assessment [14–19], the study will elicit stakeholder preferences to quantify the relative importance of DHI attributes and inform systematic evaluation strategies. By weighting multidimensional aspects based on stated preferences, the study will identify the critical factors that drive stakeholder acceptance and adoption. DCEs are a widely recognized stated preference method that quantifies choices between hypothetical alternatives differing systematically in key attributes [20]. Compared to rating or ranking techniques, the forced-choice format used in DCEs reduces cognitive burden and better mimics real-world decision-making processes [21–23]. Extensive methodological guidance and empirical validation further support the application of DCEs in health research [24–28]. DCEs are well suited for capturing trade-offs between intervention attributes, they face limitations when dealing with large attribute sets. To overcome these limitations, BWS Type 1 provides a complementary approach that enables a full-scale ranking across decision domains.

The final study framework will support the informed integration of DHIs into healthcare systems and provide actionable insights for decision-makers, developers, and policymakers based on stakeholder preferences. The specific objectives of the study are:

- To define and operationalize value dimensions relevant to the evaluation of DHIs, based on a systematic review, qualitative interviews, and expert input.
- To identify decision-relevant attributes across these dimensions represent the key drivers of stakeholder preferences.
- To assess the relative importance of these attributes using stated preference methods, with a focus on quantifying trade-offs and priority structures.
- To estimate stakeholders’ willingness to pay (WTP) for improvements in different DHI attributes, providing monetary valuations.
- To evaluate stakeholders’ willingness to invest time (WTV) in using DHIs, capturing non-monetary costs and behavioral constraints.
- To analyze heterogeneity in preferences across stakeholder subgroups, considering factors such as prior experience, demographics, and health status.

By addressing these objectives, the study will generate a comprehensive preference-based framework for evaluating DHIs and contribute to the development of more stakeholder-centered digital health strategies.

### Target populations and sampling

The study will include two stakeholder groups:

#### General population (*N* = 4,000)

Recruited via quota sampling based on demographic variables such as age, gender, and region. Participants represent current and potential users of DHIs and are not required to report on specific medical conditions. Respondents will be post-classified according to self-reported health status (e.g., acute, chronic, or healthy).

#### Healthcare providers (*N* = 250–300)

Recruited through collaboration with the Association of Statutory Health Insurance Physicians in Lower Saxony (KVN) and, if necessary, through additional professional recruitment channels. Eligible participants include licensed physicians and health care professionals actively engaged in patient care.

Eligibility criteria include being at least 18 years of age, residing in Germany, and having sufficient German language proficiency to complete the survey independently.

Participants with incomplete surveys or implausible response patterns may be excluded from the analysis. Potential indicators for exclusion include straight-lining (i.e., selecting the same response option across all choice tasks regardless of content), unrealistically short completion times based on empirical response benchmarks, inconsistent answering behavior such as dominance test failures, and incoherent or irrelevant entries in open-text fields. Exclusions may result from individual criteria or from combinations thereof. A comprehensive quality control procedure will be implemented to identify low-quality responses, and all exclusion decisions will be documented transparently.

### Selection of attributes and levels

Attribute and level identification for the DCE [29] followed a four-step process: systematic review, review of reviews, qualitative interviews, and expert validation. This approach ensured comprehensive coverage of factors critical to DHI evaluation.

#### Systematic review on value assessment frameworks

The systematic review identified and synthesized evaluation criteria from existing value assessment frameworks (VAFs) to inform the definition of key attributes for DHI assessment. The extracted dimensions directly guided the development of the conceptual framework used in DCE. The review focused on developing a complete list of value dimensions, including both general and specific components relevant to DHI evaluation.

The review followed the PRISMA guidelines (Preferred Reporting Items for Systematic Reviews and Meta-Analyses) to ensure methodological rigor [30]. The search identified 2,104 records from electronic databases, and 2,061 titles and abstracts were screened after removing duplicates. Following the exclusion of irrelevant reports and citation searches, 97 relevant sources were included in the final analysis.

The review demonstrated that the value of DHIs requires a multidimensional evaluation. It identified four key dimensions:

- **Impact on the Subject**: Examining user-related value contributions, such as clinical benefits and behavior changes [3, 31].
- **Impact on the Interaction**: Assessing the quality and efficiency of user-DHI interactions, including user-friendliness [32, 33].
- **Impact on the System**: Evaluating supporting infrastructure and mechanisms, such as interoperability and cost efficiency [34, 35].
- **Impact on Society**: Considering regulatory frameworks, long-term societal effects, sustainability, and societal acceptance [36].

The findings highlighted that existing assessment approaches are often fragmented and overlook essential aspects. This review addresses these gaps by promoting a multidimensional framework for evaluating DHIs, providing a foundation for future research to improve their efficiency and acceptance.

#### Review of review on effectiveness and outcomes in DHI evaluation

The objective of this review of reviews was to identify value criteria and metrics for evaluating both patient-facing and provider-facing DHIs. As DHIs have become increasingly integrated into healthcare, traditional clinical evaluations have expanded to include user, system, and societal dimensions alongside clinical utility. This review systematically extracted and categorized evaluation criteria across clinical, user, system, and societal dimensions, providing the empirical foundation for selecting and defining the attributes assessed in the DCE. The criteria identified were subsequently consolidated into candidate attributes, which were further refined through qualitative interviews and expert discussions.

The search followed PRISMA guidelines and initially identified 460 records. After removing 46 duplicates and excluding 268 irrelevant records based on title and abstract screening, 151 full-text articles were assessed. Following application of pre-specified inclusion and exclusion criteria, 147 articles were included in the final analysis.

The review identified a wide range of value criteria and metrics, categorized across the four key dimensions of value creation and structured as attributes. See Table 1.

#### Qualitative interviews

The qualitative interviews refined and validated the attributes identified during the literature reviews. Ten participants representing diverse perspectives were included to assess the relevance and importance of the attributes and their alignment with real-world preferences.

Recruitment followed two approaches: five participants were recruited through an unbiased third-party provider without quotas, and five were selected via professional and academic networks in health sciences. This included students, young healthcare professionals, and members of a senior citizens’ network to ensure broad representation.

Participants discussed how attributes across the four value dimensions impacted their lives and influenced their health intervention preferences. A card-sorting exercise was used, asking participants to rank the attributes by perceived importance.

This process identified the most relevant and actionable attributes for inclusion in the final analysis. Leadership-level participants contributed additional insights into system-related attributes compared to participants without professional healthcare experience. Overall, the interviews enhanced understanding of stakeholder preferences and informed the final DCE attribute set.

#### Expert discussions

In the fourth step, the research team conducted a structured expert workshop to validate and refine the preliminary dimensions and attributes developed through the prior literature review and qualitative interviews. Six experts contributed, representing a broad spectrum of institutional perspectives, including statutory health insurance, regional physician networks, academic public health research, digital health strategy, and health system governance. The workshop followed a predefined moderation framework. The team presented the preliminary conceptual model and attribute set, which served as the basis for a guided discussion focused on the relevance, clarity, and completeness of the proposed elements. While the session was not audio-recorded or transcribed, detailed written summaries captured the key points of the discussion. Attribute adjustments were made based on group-level consensus, most notably the expansion of the societal dimension to include long-term public health outcomes and sustainability considerations. This step strengthened the conceptual robustness of the model and confirmed its ability to represent value across individual, interactional, system, and societal levels.

#### Decision models of four value dimensions

A decision model defines the set of attributes relevant to the preference elicitation process in DCE. In this study, four decision models were developed based on literature, interviews and discussions. Each model reflects a specific value dimension, capturing stakeholder-relevant value contributions across the four domains: Impact on the Subject (e.g., clinical benefits, behavior changes), Impact on the Interaction (e.g., usability and interaction quality), Impact on the System (e.g., interoperability, efficiency, cost), and Impact on Society (e.g., sustainability, regulation, societal acceptance). A final set of 31 descriptive attributes were categorized into the dimensions as presented in Table 1 and Additional file 1. Each attribute was accompanied by a textual description and an icon to support visual comprehension.

Attribute levels were constructed using a standardized goal attainment framework, defining incremental improvements relative to a pre-digitalization status quo. Each attribute included five levels of achievement: 0%, 25%, 50%, 75%, and 100%. This consistent level structure supported comparability across attributes and value dimensions.

For visual differentiation of achievement levels, a viridis color scale [37], was applied to the associated pictograms. See Table 1. The viridis scale was selected to ensure accessibility for participants with color vision deficiencies and to avoid negative connotations associated with traditional color gradients such as red or yellow. This approach supported a perceptually uniform, inclusive survey design.

**Table 1:**
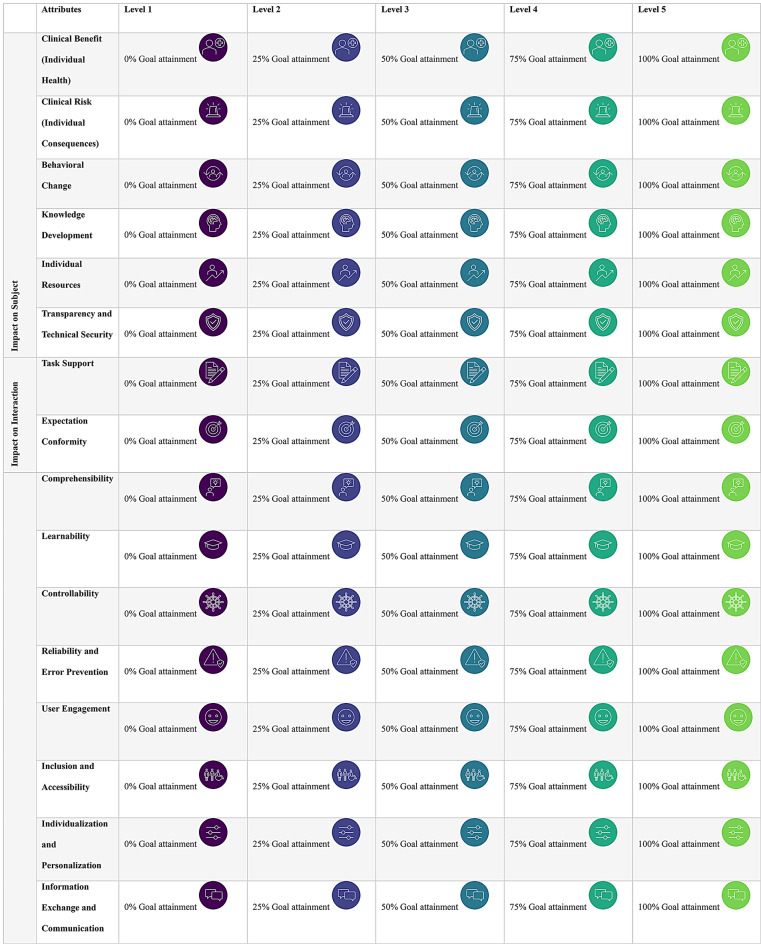

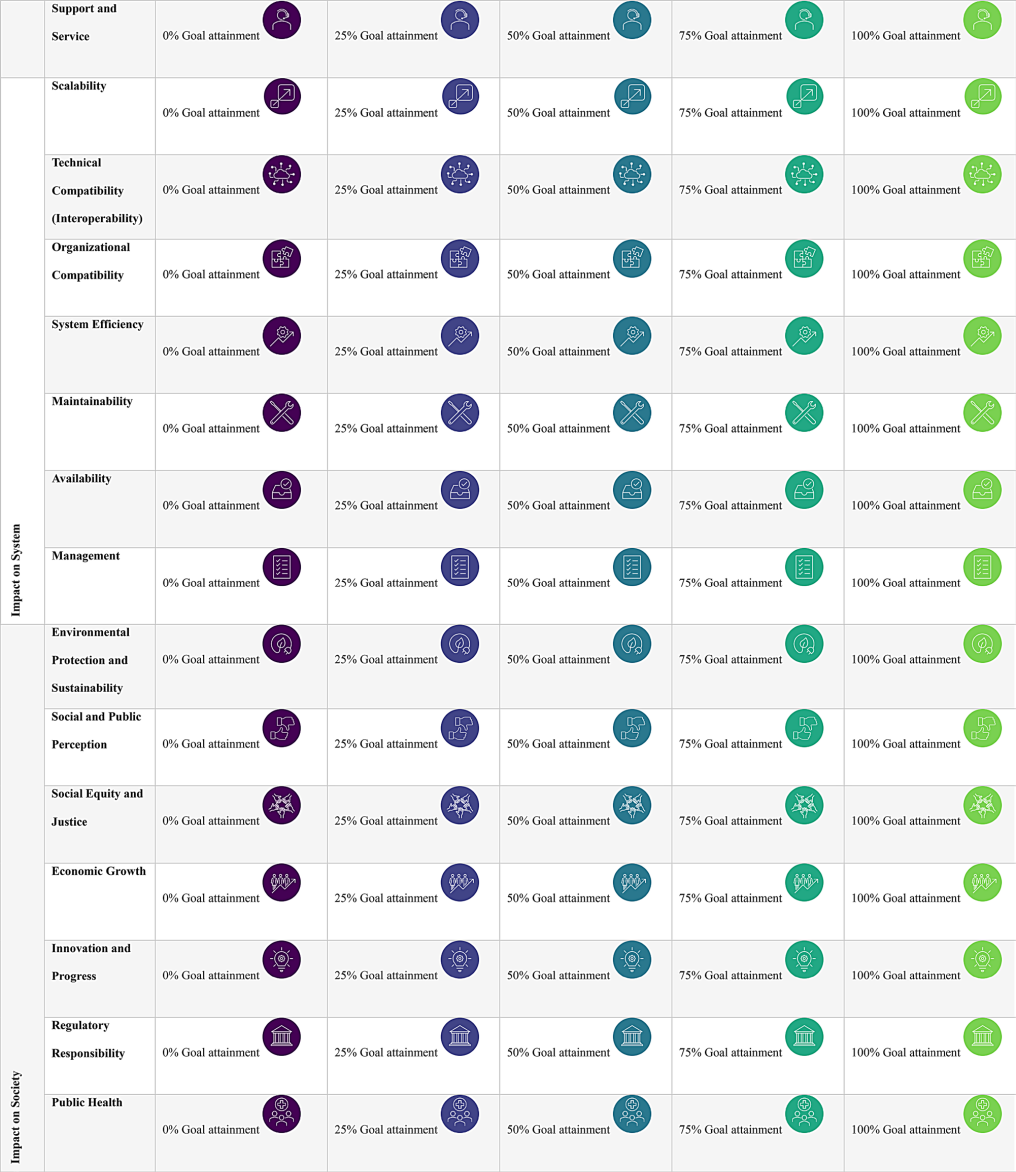
Overview of attributes and levels

#### Continuous attributes to enhance cross-dimensional comparability

Continuous attributes were incorporated into the DCE design to enhance comparability across the four decision models by establishing a consistent scaling framework. Modeling key trade-off variables such as WTP, WTV, and initially health insurance expenditures enable robust cross-model and cross-attribute analysis.

Out-of-Pocket Costs (WTP) captures the maximum monetary amount participants are willing to pay for an intervention. As a continuous variable, WTP facilitates the evaluation of price-performance trade-offs and provides an interpretable monetary scaling across scenarios.

Individual Time Investment quantifies the daily time burden associated with DHI use, including setup, engagement, and training time. Time is modeled consistently across DCEs to support comparability in marginal value analyses.

Health Insurance Expenditures was initially considered to assess systemic financial impacts from a payer perspective. However, pre-testing revealed that respondents deprioritized this attribute, leading to its exclusion from the final model.

To ensure valid interpretation of cost attributes, a scope test will be implemented [38]. The maximum level of the monthly cost attribute was defined differently for two subsamples: for half of the participants, the highest cost level will be €10 higher than in the standard version. This design allows for a controlled assessment of participants’ sensitivity to variations in cost magnitude. Participants will be randomly assigned to one of the two cost range conditions prior to survey completion.

Attribute levels are detailed in Table 2.

**Table 2:**
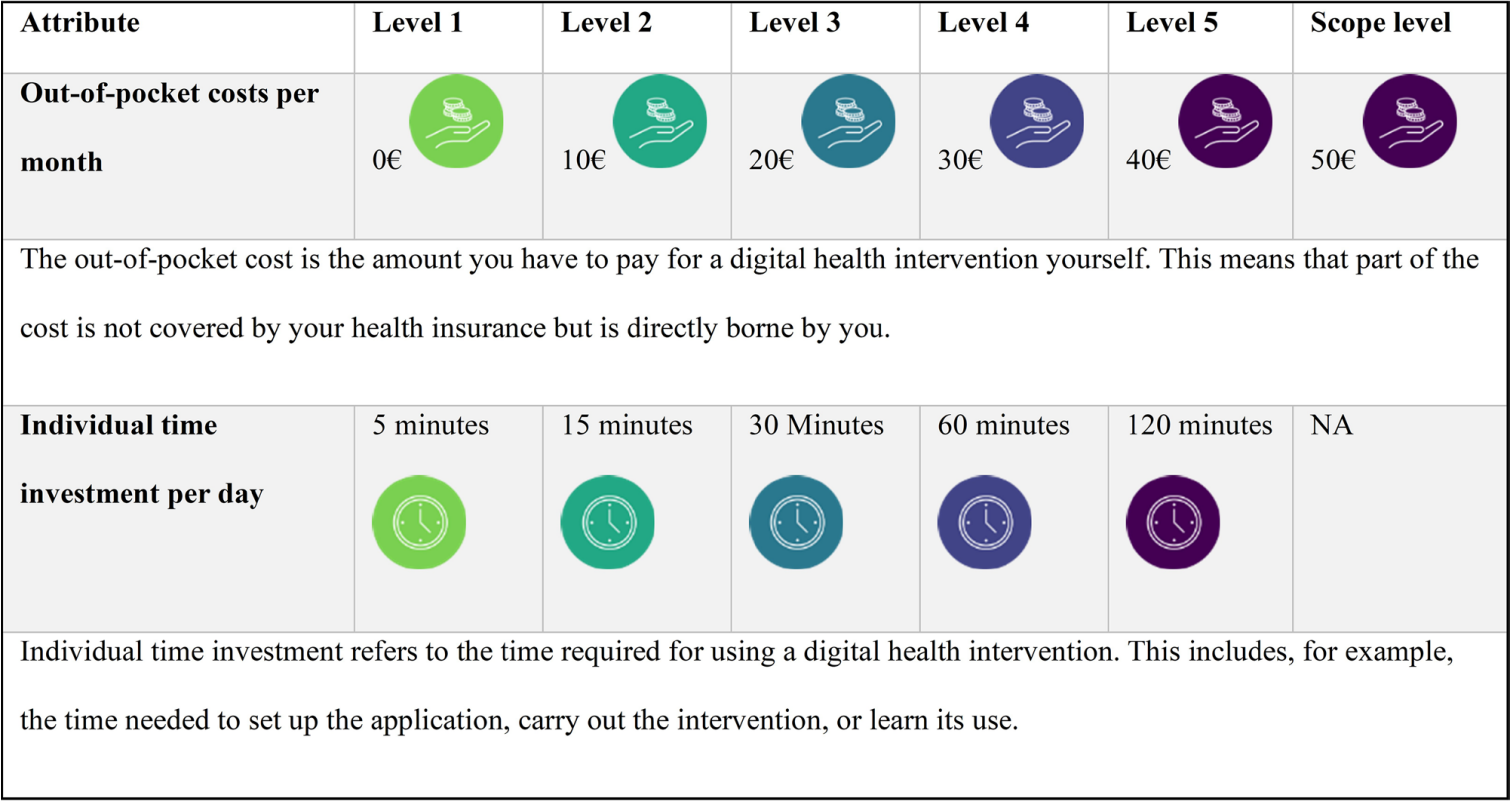
Description of continuous attributes

### Survey instrument

**Fig. 1:**
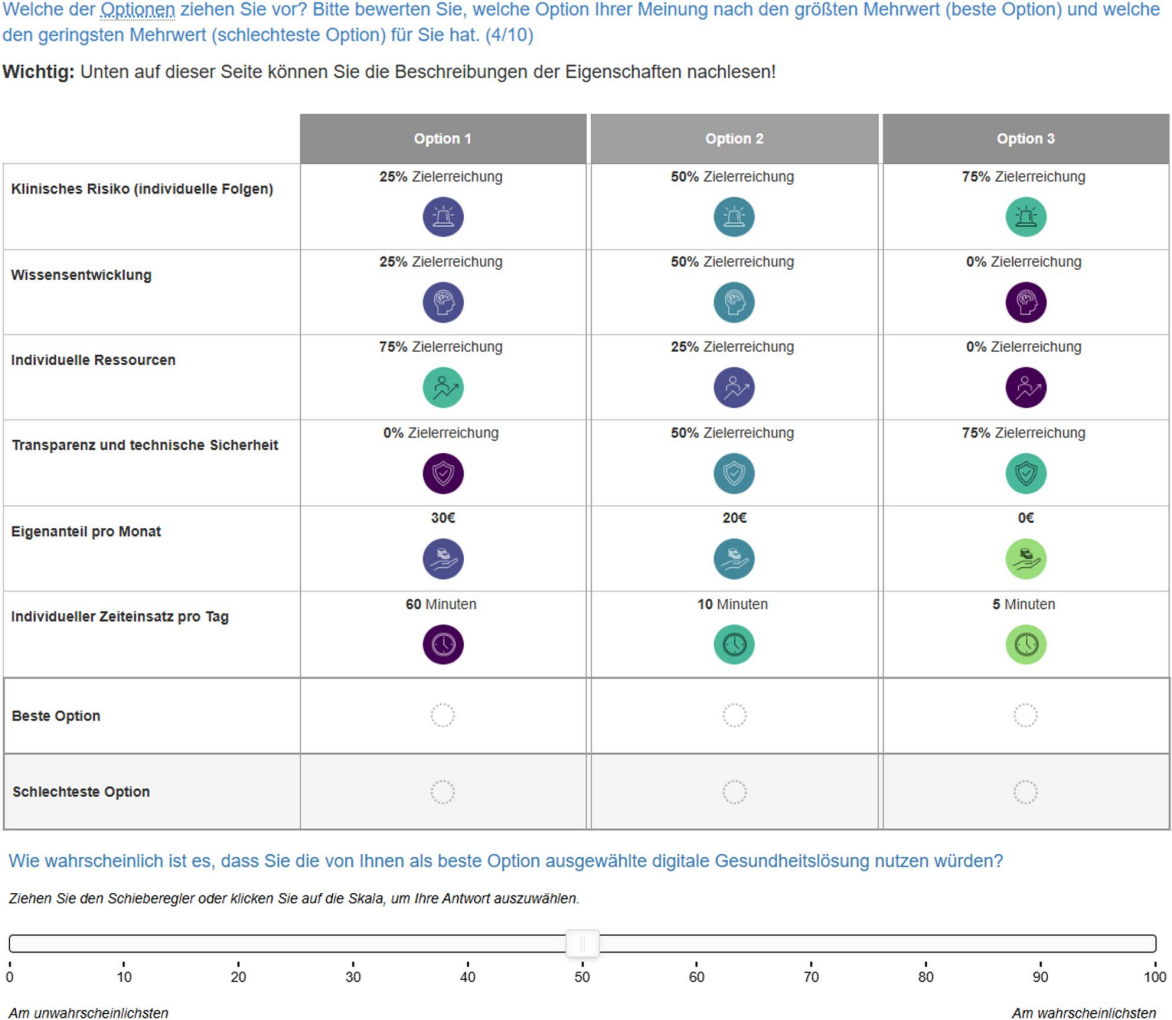
Example of a full task including additional scale, subject dimension

The survey instrument was developed to implement four independent DCE modules, each corresponding to one of the four decision models. These modules were fully integrated into a single online survey. Each participant will be randomly assigned to one DCE module to reduce cognitive burden and maintain the recommended limit of attributes per respondent [39]. Each DCE module presents nine randomized choice tasks and one dominance task to evaluate internal response consistency [25, 39]. This structure minimizes cognitive fatigue while ensuring sufficient variation to estimate reliable preference models. In each task, respondents will select the most and least preferred DHI option [40], following a best-worst forced-choice format. A forced-choice approach was applied without an opt-out option, as the study aimed to maximize information on relative preferences rather than predict actual uptake behavior. Figure 1 illustrates a sample choice task.

### Partial profile design

To manage the large number of attributes and reduce cognitive burden, the study applied a partial profile design in the DCEs [41–43]. In each choice task (per decision model), participants are shown only a subset of the total attributes. However, across the full set of tasks, each participant will encounter all attributes. The visible attributes vary between tasks and are presented at different goal attainment levels. Attributes not shown in a given task are held constant at a goal attainment level of 75%, as explained to participants during the task introduction.

Per choice task, the Subject model displays 4 of 6 descriptive attributes, the Interaction model 5 of 11, and both the System and Society models 4 of 7, each supplemented by the two continuous attributes.

This approach reduces cognitive burden while maintaining attribute coverage and interpretability. This selective presentation mirrors real-world scenarios, where not all features are simultaneously salient for example, secure data exchange is critical for electronic health records, while user engagement matters more for behavior-change apps.

To preserve transparency, the survey displayed a complete attribute list in every choice task.

The use of a partial profile design allowed the study to balance realism, cognitive feasibility, and estimation efficiency. Detailed methodological specifications for attribute distribution, choice task construction, and randomization procedures are provided in Additional file 2.

### Experimental design for the partial profile approach

The partial profile design was developed through a structured multi-step process to optimize cognitive manageability and maximize design efficiency.

First, a candidate set of choice tasks was generated to serve as the input for final experimental optimization in Ngene. The candidate set included a large number of hypothetical choice tasks with predefined attribute-level overlaps. According to the Ngene User Manual, the initial candidate set does not need to be optimized, as Ngene automatically selects suitable choice tasks during the final design generation phase.

In the finalized partial profile design, two attributes remained visible across all alternatives in each choice task, with varying attribute levels. The remaining attributes were partially displayed, ensuring that each task presented only a subset of varying attributes to reduce cognitive burden.

### Generation of the candidate set: two methods

#### Method 1: Stata

A random experimental design was first generated in Stata, allowing attribute-level overlaps between alternatives without initial restrictions. In a subsequent step, all choice tasks where an attribute exhibited identical levels across all three alternatives were removed. Overlaps between two alternatives were permitted to maintain realism while simplifying task complexity. Systematic overlaps were then introduced by randomly fixing the same attribute level across alternatives for selected attributes, ensuring a balanced representation of constant and varying attributes.

#### Method 2: Ngene

Parallel to the Stata approach, a fractional factorial design was generated in Ngene using constraint specifications (the reject; property) to prohibit complete attribute-level overlaps across alternatives. After this initial design phase, systematic attribute overlaps were randomly introduced to mirror the balancing principles applied in Method 1, enhancing consistency between the two generation strategies.

#### Selection and finalization of the candidate set

After both candidate sets were constructed, a random selection was performed to reduce the set to 2,000 choice tasks to ensure computational feasibility during the optimization process in Ngene. From this reduced candidate set, Ngene selected the most suitable choice tasks according to efficiency and balance criteria.

#### Processing and preparation of the final design

The finalized choice tasks were structured and formatted in Stata to ensure a standardized, organized layout. The completed design was then saved in Excel format for seamless integration into the survey platform and subsequent fieldwork preparation.

We ultimately selected the design generated by Ngene due to its superior efficiency measures.

### Combining DCE and BWS to handle a large attribute number

Given the high total number of attributes and their distribution across four distinct DCE modules, a methodological approach inspired by Zhang et al. [44] was adopted. To compensate for missing data on attributes not included in each DCE, a BWS task type 1 was incorporated in which participants ranked all 31 attributes by their relative importance. This approach allowed inference of preferences across all attributes while maintaining a manageable cognitive load for respondents. To enable comparability between BWS importance scores and DCE utility differences, a scaling factor (𝑓(𝜃)) was calculated by leveraging attributes common to both methods.

The relative importance 𝐼_𝐾_ of an attribute 𝐾 in the BWS data was assumed to be proportional to the utility difference (Δ𝛽_𝐾_) in the DCE data, where Δ𝛽_𝐾_ = 𝛽_𝐾,max_ − 𝛽_𝐾,min_, and 𝑓(𝜃) is the estimated adjustment factor.

Combined estimation was performed using a joint likelihood function: 𝐿 = 𝐿_DCE_ + 𝜆 ⋅ 𝐿_BWS_, where 𝜆 represents the scale adjustment parameter accounting for differences in response variance between the elicitation formats.

By pooling the data into a joint model, the study aims to generate robust preference estimates across all attributes, overcome the traditional limitations of DCEs in handling large attribute sets, and improve the internal consistency and richness of the findings. This integrated approach is expected to provide a more comprehensive understanding of stakeholder preferences in digital health decision-making.

### Survey framing and additional design elements

In addition to the experimental structure of the choice tasks, the survey was carefully framed.

#### Additional data and questions

Metadata (or paradata) providing additional information about respondents will be collected throughout the survey. These data include time spent on the overall survey and on individual questions, as well as dropout patterns. At the beginning of the survey, screening questions based on inclusion criteria will be used to determine participant eligibility.

To approximate real-world acceptance, participants will also answer a pre-DCE question:

How likely would you be to actually use your chosen option?

Beyond choice data, the survey will collect independent variables that may influence decision-making, including prior DHI experience, current health status, and key sociodemographic factors for subgroup analysis.

#### Explanations and contextualization

At the beginning of the survey, conceptual background based on the World Health Organization’s classification of DHIs [2] will be provided to support informed decision-making. Respondents will be introduced to four categories of DHIs: (1) patient and caregiver support (e.g., health promotion apps), (2) healthcare provider tools (e.g., treatment planning systems), (3) health management systems (e.g., resource planning), and (4) data services (e.g., data collection and analysis tools).

Before starting the choice tasks, participants will receive standardized definitions of all attributes and levels, supported by pictograms to enhance visual comprehension (see Table 1*)*. Participants will first be introduced to the specific value dimension to which they have been randomly assigned. This introduction includes a brief textual description of the dimension and detailed definitions of the associated attributes.

Two key concepts will be explained in advance to ensure consistent interpretation of the choice tasks. The ‘reference point’ (status quo) will be defined as the pre-digitalization baseline against which improvements are evaluated. ‘Goal attainment’ will be introduced as the level of improvement achieved relative to this baseline and operationalized through a structured percentage-based scale. To enhance intuitive understanding, a school grading analogy was described:

- **0% – Insufficient**: No progress; requirements unmet.
- **25% – Sufficient**: Minimal improvement; significant deficiencies remain.
- **50% – Satisfactory**: Moderate improvement; around half of the goal achieved.
- **75% – Good**: Substantial improvement; minor gaps persist.
- **100% – Very good**: Full or surpassing goal achievement; no major deviations.

A sample choice task will then illustrate the structure and logic of the upcoming DCE tasks. All attribute descriptions relevant to the assigned dimension will be displayed again below each individual choice task.

After completing the DCE module, participants will receive short descriptions of the remaining three value dimensions. This step provides conceptual continuity and prepares respondents for the subsequent BWS Type 1 task, which includes all 31 attributes across all dimensions. During the BWS task, participants will see the full set of attribute descriptions to ensure informed comparisons across domains.

### Pre-testing and pilot-testing

The survey instrument was pretested with six individuals recruited from the study team and affiliated networks, including students, senior citizens, and members of the general public. The pretest applied the Think-Aloud method, instructing participants to verbalize their thoughts while completing the draft version of the survey. Their feedback on text clarity, headings, question phrasing, examples, attribute descriptions, visualizations, and choice sets provided critical input for refining the instrument. All attribute levels and visual elements were systematically tested to ensure clarity, plausibility, and interpretability.

In-depth probing focused on participants’ understanding of the choice tasks and their perceived difficulty in making trade-offs. Based on the insights gained, the research team revised and internally reviewed the survey. The pretest also assessed whether participants could complete all four DCE modules. Given the cognitive burden, it was determined that each respondent should be assigned to a single DCE variant.

The survey instrument was then reviewed and approved by the Ethics Committee of Hochschule Neubrandenburg (HSNB/218/24).

Following the pretest, the decision was made to condition pilot testing of the final instrument on initial field performance. A pilot test with up to 200 participants (50 per DCE module) is planned. The pilot data will first be analyzed for internal validity using a Conditional Logit Model.

### Sample size

The study aims to recruit a total of 4,250–4,300 participants across two stakeholder groups which will be randomly assigned to one DCE module. Sample size calculations were guided by Orme’s rule-of-thumb for DCEs, assuming an average task complexity of 9 choice sets, 3 alternatives, and 6–7 active attributes per task [45]. These estimates were validated through simulation-based standard error testing for key attribute coefficients, targeting a maximum standard error of 0.05 for main effects. A minimum of 500 participants per DCE module is required to ensure sufficient power to estimate main effects.

To enable robust exploration of subgroup differences based on health experience, professional background, and familiarity with digital health interventions, the study aims to recruit a minimum of 1,000 respondents per module.

### Recruitment strategy

Participants for study population 1 (general population) will be recruited through a professional online panel provider. The provider maintains a pre-profiled panel and applies controlled sampling procedures. Quota-based stratification will ensure representativeness by age, gender, education, and state, based on official data from the German Federal Statistical Office (Destatis). Duplicate entries will be minimized through technical safeguards such as cookie tracking and redirect validation. Participants will receive an incentive only upon full completion of the survey.

Participants for study population 2 (healthcare providers and health care professionals) will be recruited via the established network of the Association of KVN and additional professional recruitment channels. No incentives will be provided for this group. Only physicians currently practicing in Germany or health care professionals affiliated with the German health care system will be eligible to participate.

To prevent fraudulent or automated participation, several quality control mechanisms are embedded in the survey. These include a hidden item not visible to human respondents but detectable by bots, as well as interactive item formats such as matrix questions, drag-and-drop elements, and visual analogue scales.

### Data management and statistical analysis

Data will be managed and analyzed according to predefined quality and modeling procedures. Additional technical details including simulation parameters, variable coding schemes, and full model specifications are provided in Additional file 3.

#### Statistical analysis

The primary analysis of DCE data will use Conditional Logit Models to estimate aggregate preferences under the assumption of homogeneous choice behavior, in line with established DCE guidelines [45–47].

Mixed Logit Models (also referred to as random parameter logit models) will subsequently be applied to account for unobserved preference heterogeneity at the individual level by allowing preference parameters to vary across respondents [47, 48].

To further explore heterogeneity, Latent Class Analysis will be used to identify discrete segments within the sample that share similar choice patterns. Unlike the mixed logit model, which assumes continuous preference variation, the latent class model assumes the existence of qualitatively distinct subgroups with shared preference structures [49, 50].

BWS responses will be analyzed using count analysis and multinomial logit models to estimate the relative importance of each attribute across the full set [44]. DCE and BWS data will be integrated using a joint likelihood framework, applying scale adjustment parameters to harmonize utility magnitudes across elicitation formats.

Attributes will primarily be coded using effects coding, enabling interpretation relative to grand means. Missing data will be handled through case-wise deletion for incomplete choice sets, with additional sensitivity analyses planned to assess the robustness of results.

All analyses will be conducted using Stata (Version 17.0, StataCorp) [51]. Model fit will be evaluated using information criteria (Akaike Information Criterion, Bayesian Information Criterion) and likelihood ratio tests, where applicable.

Data processing and analysis will adhere to GDPR regulations and institutional data protection standards, including secure storage, anonymization, and restricted access.

#### Null hypotheses

This study will test the following null hypotheses, each corresponding to one of the four DHI value dimensions. These hypotheses focus on the impact of attribute level variations on respondents’ choice behavior and will be evaluated using statistical methods:

- **H₀₁**: Variation in attribute levels related to the Subject dimension (e.g., clinical outcomes, quality of life) does not significantly influence respondents’ choices.
- **H₀₂**: Variation in attribute levels related to the Interaction dimension (e.g., usability, accessibility) does not significantly influence respondents’ choices.
- **H₀₃**: Variation in attribute levels related to the System dimension (e.g., efficiency, interoperability) does not significantly influence respondents’ choices.
- **H₀₄**: Variation in attribute levels related to the Society dimension (e.g., sustainability, equity) does not significantly influence respondents’ choices.

#### Data security, validity, and reliability

To ensure data integrity in this study, several quality assurance steps will be implemented, including end-to-end testing of the survey infrastructure, test data tabulation prior to fielding, validation of the experimental design, and verification of all statistical analysis scripts. Additional quality indicators will be collected during fielding, such as total survey completion time for the entire web survey, choice of dominant alternative in the input choice set, attributes non-attendance and straight-lining (i.e., choosing the same response option in each choice set) [52].

To detect fraudulent or automated responses [53], a set of behavioral indicators will be analyzed post hoc. These include the total completion time, which will be benchmarked against expected reading and response durations; the time of participation, with particular attention to atypical nighttime activity; and response patterns, such as repetitive or invariant choices across DCE or BWS tasks. In addition, open-ended responses will be reviewed for semantic coherence and plausibility. These combined measures aim to ensure high data quality and to exclude non-human or inattentive response behavior from the final analysis.

## Discussion

This study protocol outlines the development and design of a HPR study aimed at evaluating stakeholder preferences for DHIs in the German healthcare context. By employing a DCE methodology, the study addresses the multidimensional nature of DHI evaluation, encompassing clinical, technological, economic, and societal factors. The inclusion of four distinct decision models, each aligned with a specific value dimension, provides a comprehensive approach to capturing user and provider preferences and guiding the integration of DHIs into practice.

The study framework incorporates continuous attributes, such as WTP and WTV, to enhance comparability across models and allow monetary interpretation of preference structures. The systematic selection of attributes and levels, grounded in literature reviews, qualitative interviews, and expert discussions, establishes a strong foundation for DCE design. The user-centered approach featuring clear attribute definitions, visual support, tutorials, and extensive pretesting further strengthens the reliability and validity of the survey instrument.

Beyond advancing methodological approaches in health preference research, this protocol contributes to the broader discourse on digital health adoption by systematically capturing the heterogeneity of preferences across diverse stakeholder groups. The findings are expected to inform policymakers, developers, and healthcare providers about the critical drivers and barriers to DHI acceptance and integration, thereby addressing existing gaps in the evaluation and implementation of digital health technologies.

The reporting of study results will follow established guidelines for discrete choice experiments, including the ISPOR ESTIMATE and DIRECT checklists, to ensure methodological rigor and transparency [45, 54].

### Why hypothesis testing matters in a DCE context

While DCEs are primarily designed to explore trade-offs and preference structures, explicitly testing null hypotheses adds methodological rigor by formally evaluating whether variations in attribute levels meaningfully influence choice behavior. Testing the impact of value dimensions (subject, interaction, system, and society) ensures that the observed choices reflect systematic preferences rather than random variation or cognitive noise. This approach enhances the interpretability and validity of the study results and supports the generalizability of findings for policy, clinical, and technological decision-making.

### Limitations

Despite its strengths, this study has several limitations. First, although efforts were made to minimize cognitive burden by assigning participants only a subset of choice tasks, some respondents may still find the DCE cognitively demanding, which could affect data quality. Second, reliance on hypothetical scenarios may not fully reflect real-world decision-making behaviors. Third, the perceived reference point (status quo) might vary across participants, potentially complicating the interpretation of goal attainment levels. However, providing detailed explanations and maintaining consistent decision contexts within the survey is expected to mitigate these risks.

Continuous attributes offer an important advantage by enabling direct comparisons across different DCEs. Estimating marginal WTP (€/time unit or €/attribute unit), preference elasticities, and implicit attribute valuations allows for normalization and scaling across experiments. Nonetheless, challenges exist, including increased cognitive burden when assessing continuous trade-offs, potential scaling inconsistencies between experiments, and the influence of external factors such as cultural context. Careful normalization and interpretation will be required to ensure comparability. Despite these challenges, continuous attributes remain a robust tool for capturing nuanced stakeholder preferences.

One potential limitation of the partial profile design is variability in how participants interpret non-displayed attributes. Research suggests that including attributes with identical levels across alternatives (level overlap) can enhance choice consistency and decision accuracy [55, 56]. However, given the context-specific relevance of many DHI attributes, level overlap was deemed unsuitable for this study, as it would have unnecessarily prolonged choice tasks and increased cognitive burden. Previous research supports the appropriateness of partial profile designs for managing complexity while maintaining realism [41, 57]. Careful experimental design strategies, as recommended by Kessels et al. [42, 43], were applied to optimize estimation efficiency. A detailed discussion of methodological limitations and design considerations is provided in Additional file 2.

## Conclusion

This study protocol presents a comprehensive framework for developing and implementing an HPR study focused on evaluating DHIs. By systematically addressing the multidimensional value of DHIs through a robust DCE design, the study aims to generate actionable insights into stakeholder preferences and decision-making processes. The findings are expected to support the development of evidence-based strategies for evaluating, prioritizing, and integrating DHIs into healthcare delivery. Ultimately, the study seeks to contribute to more patient-centered, efficient, and widely accepted digital health solutions.

## Supplementary Information

Below is the link to the electronic supplementary material.

Supplementary Material 1

Supplementary Material 2

Supplementary Material 3

## Data Availability

No datasets were generated or analysed during the current study.

## Declarations

### Study status

Participant recruitment began in June 2025 and is currently ongoing. The recruitment phase is expected to be completed by 30 September 2025.

### Author‘ information

AF is a research associate in Health Economics and Health Management at Hochschule Neubrandenburgand a doctoral candidate at the University of Greifswald, focusing on health preference research and value assessment frameworks. AS is a research associate with expertise in quantitative methods for preference research andthe design and analysis of choice experiments. AM is Professor of Health Economics and Health Managementat Hochschule Neubrandenburg, specializing in decision analysis, health economic evaluation, and preferencemeasurement.

## Abbreviations

KVN: Association of Statutory Health Insurance Physicians in Lower Saxony
BWS: Best Worst Scaling
DCE: Discrete Choice Experiments
HPR: Health Preference Research
PRISMA: Preferred Reporting Items for Systematic Reviews and Meta-Analyses
VAF: Value Assessment Frameworks
WTP: Willingness to Pay
WTV: Willingness to Time Invest
