# Supplementary material for: Stakeholder preferences on digital health in Germany: a health preference study protocol
Source: BMC Health Serv Res. 2026 Apr 29;26:616. doi: 10.1186/s12913-026-14216-8 (PMC13134233; doi:10.1186/s12913-026-14216-8)
Supplement: Supplementary file 1 — Supplementary Material 1 [file 12913_2026_14216_MOESM1_ESM.docx]

Additional file 1: Descriptions of each attribute, translated from original language German

| **Dimension** | **Attributes** | **Description** |
| --- | --- | --- |
| **Impact on Subject** | **Clinical Benefit (Individual Health)** | Clinical benefit describes how the digital health solution measurably improves individual health for users, e.g., through stable health values, early disease detection, or more effective treatments. The goal is to significantly promote health. |
|  | **Clinical Risk (Individual Consequences)** | Clinical risk describes how a digital health solution helps reduce potential negative health consequences, e.g., by avoiding side effects, detecting misdiagnoses, or ensuring correct application. The goal is to prevent negative health outcomes. |
|  | **Behavioral Change** | Behavioral change describes how the digital health solution promotes a healthier lifestyle, e.g., by supporting nutrition, avoiding addictive substances, or improving sleep habits. The goal is to encourage new and healthier behaviors. |
|  | **Knowledge Development** | Knowledge development describes how the digital health solution conveys understandable health knowledge, e.g., through practical and easily accessible information. The goal is to promote access to relevant knowledge and its application. |
|  | **Individual Resources** | Individual resources describe how the digital health solution saves resources such as time, money, and energy, e.g., through remote treatments or automated processes. The goal is to reduce individual effort for healthcare services. |
|  | **Transparency and Technical Security** | Transparency and technical security describe how the digital health solution provides necessary information and protects personal data, e.g., through provider information, technical specifications, and security against unauthorized access. The goal is to minimize existing security risks in healthcare and enable trustworthy use. |
| **Impact on Interaction** | **Task Support** | Task support describes how the digital health solution helps users complete tasks easily, e.g., through reminders, automatic input suggestions, or focusing on important aspects while reducing distractions. The goal is to accomplish tasks with minimal effort. |
|  | **Expectation Conformity** | Expectation alignment describes how the digital health solution meets users' expectations and habits, e.g., through familiar navigation, symbols, and content. The goal is to use understandable structures and familiar representations. |
|  | **Comprehensibility** | Comprehensibility describes how the digital health solution provides clear orientation, e.g., by offering information on current steps. The goal is to enable users to navigate easily. |
|  | **Learnability** | Learnability describes how easily the digital health solution can be understood and used, e.g., through explanations and testing options. The goal is to learn and apply new functions quickly. |
|  | **Controllability** | Control options describe how the digital health solution enables users to use it independently, e.g., when and how they utilize its functions. The goal is to allow responsible usage. |
|  | **Reliability and Error Prevention** | Reliability and error prevention describe how the digital health solution functions stably and detects and corrects input errors, e.g., through warnings or automatic corrections. The goal is to ensure a smooth experience. |
|  | **User Engagement** | User engagement describes how the digital health solution motivates users, e.g., through gamification elements or attractive design. The goal is to encourage long-term enjoyment of use. |
|  | **Inclusion and Accessibility** | Access and accessibility describes how the digital health solution is available to all users, e.g., regardless of technical, physical, or psychological abilities or cultural backgrounds. The goal is to enable barrier-free use. |
|  | **Individualization and Personalization** | Customizability and personalization describe how the digital health solution can be tailored to user needs, e.g., through manual or automatic adjustments. The goal is to enable individual offerings. |
|  | **Information Exchange and Communication** | Information exchange and communication describe how the digital health solution facilitates information sharing, e.g., with professionals or other users. The goal is to make relevant information easily accessible. |
|  | **Support and Service** | Support and service describe how the digital health solution provides individual assistance, e.g., through technical or professional support. The goal is to resolve issues and answer questions. |
| **Impact on System** | **Scalability** | Scalability and expandability describe how the digital health solution can reach more users and offer new features, e.g., by increasing user capacity. The goal is to adapt the solution to new requirements. |
|  | **Technical Compatibility (Interoperability)** | Technical compatibility describes how the digital health solution integrates with other systems, e.g., through seamless data exchange. The goal is to ensure easy integration into healthcare systems. |
|  | **Organizational Compatibility** | Organizational compatibility describes how the digital health solution integrates into organizational structures, e.g., by embedding into workflows. The goal is to enable seamless and appropriate implementation. |
|  | **System Efficiency** | System efficiency describes how the digital health solution saves resources, e.g., through better processes, reduced material consumption, and optimized personnel deployment. The goal is to improve the economic efficiency of the healthcare system. |
|  | **Maintainability** | Maintainability describes how easily the digital health solution can be maintained, e.g., through automatic updates and simple troubleshooting. The goal is to ensure smooth maintenance for providers and users. |
|  | **Availability** | Availability describes how the digital health solution enables care to be used regardless of time and place, e.g., through appropriate infrastructure or stable internet access. The goal is to ensure continuous usage. |
|  | **Management** | Management describes how the digital health solution supports processes, e.g., through data-based decisions and measure analysis. The goal is to better plan, control, and optimize processes in the healthcare system. |
| **Impact on Society** | **Environmental Protection and Sustainability** | Environmental Protection and Sustainability describe how the digital health solution conserves natural resources and promotes sustainability – for example, through paperless documentation or by avoiding travel via digital meetings. The goal is to reduce the ecological footprint. |
|  | **Social and Public Perception** | Social and Public Perception describes how the digital health solution is accepted by the public, for instance by meeting societal expectations. The goal is to foster acceptance and build a positive image. |
|  | **Social Equity and Justice** | Social Balance and Justice describe how the digital health solution promotes equal opportunities – for example, by supporting disadvantaged groups. The goal is to strengthen fairness and integration. |
|  | **Economic Growth** | Economic Growth describes how the digital health solution fosters the economy and creates jobs – for instance, through improved processes and the emergence of transformed or new fields of work. The goal is to ensure long-term economic stability. |
|  | **Innovation and Progress** | Innovation and Progress describe how the digital health solution offers new solutions – for example, through technological advancements. The goal is to create modern and future-proof structures. |
|  | **Regulatory Responsibility** | Regulatory Responsibility describes how the digital health solution meets legal, professional, and technical requirements – for instance, by complying with data protection guidelines or medical standards. The goal is to integrate the solution into the regulated healthcare system. |
|  | **Public Health** | Public Health describes how the digital health solution promotes the health of the population – for example, through preventive measures and improved access to healthcare services. The goal is to enhance the health, well-being, and productivity of society. |
