# Supplementary material for: Stakeholder preferences on digital health in Germany: a health preference study protocol
Source: BMC Health Serv Res. 2026 Apr 29;26:616. doi: 10.1186/s12913-026-14216-8 (PMC13134233; doi:10.1186/s12913-026-14216-8)
Supplement: Supplementary file 2 — Supplementary Material 2 [file 12913_2026_14216_MOESM2_ESM.docx]

Additional file 2: Partial profile design – detailed explanation

**Partial profile design**

In this study, a Discrete Choice Experiment (DCE) is conducted to investigate preferences regarding digital health interventions. Since digital health interventions exhibit a wide range of characteristics and can provide value at different levels (e.g., for the user, interaction, system, or society), four decision models were developed, each incorporating different relevant attributes. These four decision models were implemented in four separate DCEs. Each of the four decision models was extended by two continuous attributes (out-of-pocket costs and individual time investment) to allow for limited comparability between the models. The number of attributes in the decision models exceeds the recommended upper limit for choice tasks. To ensure an appropriate cognitive load for participants, a partial profile design was implemented [1-3].

The distribution of attributes within the individual DCEs is as follows:

- **DCE 1 (Value Contribution: Subject):** 6 descriptive attributes + 2 continuous attributes. In each choice task, 6 attributes are displayed, including 2 continuous attributes and 4 out of 6 descriptive attributes. The remaining 2 descriptive attributes are hidden.
- **DCE 2 (Value Contribution: Interaction):** 11 descriptive attributes + 2 continuous attributes. In each choice task, 7 attributes are displayed, including 2 continuous attributes and 5 out of 11 descriptive attributes. The remaining 6 descriptive attributes are hidden.
- **DCE 3 (Value Contribution: System) and DCE 4 (Value Contribution: Society):** Each includes 7 descriptive attributes + 2 continuous attributes. In each choice task, 6 attributes are displayed, including 2 continuous attributes and 4 out of 7 descriptive attributes. The remaining 3 descriptive attributes are hidden.

Attributes that are not displayed in a given choice task remain invisible to participants, meaning they are explicitly excluded from the decision-making process. Displaying only a subset of attributes in each choice task is beneficial for several reasons. First, it reduces participants’ cognitive burden, allowing them to focus on a smaller number of relevant characteristics without being overwhelmed by an excessive number of attributes. This facilitates better decision-making, as participants can evaluate the presented health interventions more accurately and in a more focused manner. Second, this approach reflects the actual complexity of health interventions, where not all attributes are relevant in every situation. For example, in the case of an electronic health record (EHR), aspects related to secure and transparent data exchange play a crucial role, whereas for behavior-change apps, attributes such as user engagement may be more relevant. The random selection of displayed attributes allows for testing different attribute combinations and obtaining a more realistic picture of participants' preferences. Potential interaction effects between attributes were analyzed and ruled out in preliminary studies (qualitative pilot and pretest interviews).

However, in order to still present respondents with the complete decision including all attributes, a list of all attributes was integrated into the question. It was also explained that any attributes not displayed had the same level of goal achievement across all alternatives in the choice task: *" 75% Goal Achievement – Good, substantial improvement, most of the goal achieved, minor gaps remain, but overall satisfactory."*

**Limitations of the partial profile design**

A key argument against using a partial profile design is the potential variability in how participants interpret attributes that are not shown. Jonker et al. [4, 5] demonstrated that including attributes with identical levels across all alternatives (level overlap) can improve consistency and choice accuracy in DCEs. Their study found that level overlap and color coding reduced the likelihood of random or inconsistent responses [5]. Another concern is that omitting attributes may reinforce unconscious decision heuristics [4]. However, these findings cannot be universally applied to all studies, as they strongly depend on the specific decision context.

**Advantages of the partial profile design**

Despite these concerns, the chosen design remains appropriate. Different digital health interventions have distinct value drivers, which are not always relevant for every intervention. While level overlap may be beneficial in some scenarios, it would not be suitable for this study. Additionally, visualizing omitted attributes would unnecessarily prolong the choice tasks and increase participants’ cognitive burden. A key example is provided by Chrzan [1], who highlights that partial profile designs are particularly useful when a large number of attributes is required to realistically depict the decision-making situation. He argues that reducing the number of attributes per choice task facilitates the decision-making process without losing essential information. Similarly, Norman et al. [6] confirm that partial profile designs can be employed to minimize cognitive burden, though they emphasize the need for careful implementation to avoid biases stemming from participants' interpretation of omitted attributes. Kessels et al. [2, 3] also examined various partial profile designs and recommended paying special attention to experimental design to improve the efficiency of parameter estimation.

**References**

1. Chrzan, K., *Using partial profile choice experiments to handle large numbers of attributes.* International Journal of Market Research, 2010. **52**(6): p. 827-840.

2. Kessels, R., B. Jones, and P. Goos, *Bayesian optimal designs for discrete choice experiments with partial profiles.* Journal of Choice Modelling, 2011. **4**(3): p. 52-74.

3. Kessels, R., B. Jones, and P. Goos, *A comparison of partial profile designs for discrete choice experiments with an application in software development.* 2012.

4. Jonker, M.F., et al., *Effect of level overlap and color coding on attribute non-attendance in discrete choice experiments.* Value in Health, 2018. **21**(7): p. 767-771.

5. Jonker, M.F., et al., *Attribute level overlap (and color coding) can reduce task complexity, improve choice consistency, and decrease the dropout rate in discrete choice experiments.* Health economics, 2019. **28**(3): p. 350-363.

6. Norman, R., et al., *Issues in the design of discrete choice experiments.* The Patient-Patient-Centered Outcomes Research, 2019. **12**: p. 281-285.
