# Supplementary material for: Stakeholder preferences on digital health in Germany: a health preference study protocol
Source: BMC Health Serv Res. 2026 Apr 29;26:616. doi: 10.1186/s12913-026-14216-8 (PMC13134233; doi:10.1186/s12913-026-14216-8)
Supplement: Supplementary file 3 — Supplementary Material 3 [file 12913_2026_14216_MOESM3_ESM.docx]

**Additional file 3: Technical analysis plan**

**Overview and objectives of the analysis**

The purpose of this analysis plan is to outline the statistical methods used to estimate stakeholder preferences for digital health interventions (DHIs). The objectives of the analysis are to (1) quantify the relative importance of decision-relevant DHI attributes, (2) estimate trade-offs between competing features, (3) calculate willingness to pay (WTP) and willingness to invest time (WTV), (4) explore preference heterogeneity across stakeholder groups, and (5) integrate data from discrete choice experiments (DCEs) and best-worst scaling (BWS) exercises into a unified, preference-based evaluation framework.

**Data preparation and quality assurance**

All survey responses will be subjected to rigorous quality checks prior to analysis. Participants must complete all assigned DCE and BWS tasks and correctly answer the dominance task to be included in the final dataset. Cases showing straight-lining behavior or implausibly short completion times will be flagged and reviewed. Attribute non-attendance patterns will be examined descriptively and used in sensitivity analyses. Missing data is expected to be minimal due to the forced-choice format; any incomplete responses will be excluded via listwise deletion.

**Discrete Choice Experiment modeling**

The primary modeling approaches for the DCE data include:

- Conditional Logit Model for estimating average preferences across the sample.
- Mixed Logit Model (random parameters logit) to account for unobserved preference heterogeneity.
- Latent Class Logit Model to identify discrete subgroups with systematically different preference structures.

DCE attributes will be effects-coded or dummy-coded as appropriate. Continuous variables include:

- Out-of-pocket costs (€/month),
- Time investment (minutes/day).

Interactions between cost/time variables and respondent characteristics (e.g., DHI usage, age, profession) may be tested in exploratory models. Model fit will be assessed using log-likelihood, Akaike Information Criterion (AIC), and Bayesian Information Criterion (BIC).

**Best-Worst Scaling (Type 1) modeling**

The BWS component consists of repeated tasks in which participants select the most and least important attributes from balanced subsets. Data will be analyzed using:

- Simple count analysis (best-minus-worst score per attribute),
- Multinomial logit models for repeated best-worst responses,
- Utility estimation of each attribute’s relative importance.

This allows full attribute ranking and complements the partial exposure in DCE tasks.

**Integration of DCE and BWS data**

To leverage both methods, a joint likelihood model will be used to align DCE and BWS outputs. A scaling factor will be calculated to adjust the difference between BWS importance scores and DCE utility differences. This process uses shared attributes as anchors to generate a consistent preference scale:
The relative importance 𝐼_𝐾_ of an attribute 𝐾 in the BWS data was assumed to be proportional to the utility difference (Δ𝛽_𝐾_) in the DCE data, where Δ𝛽_𝐾_ = 𝛽_𝐾,max_ − 𝛽_𝐾,min_, and 𝑓(𝜃) is the estimated adjustment factor.
Additionally, a scale adjustment parameter (λ) will be included in the model to account for differences in response variance between the two elicitation methods.

**Subgroup and heterogeneity analysis**

Preferences will be explored across predefined subgroups:

- General population vs. healthcare providers,
- Frequent vs. infrequent DHI users,
- Respondents with acute, chronic, or no current health conditions.

Subgroup differences will be examined through stratified models and interaction terms. Latent class analysis will be conducted to uncover unobserved preference segments. Model fit for 2–6 class solutions will be evaluated using BIC, entropy, and posterior probabilities. Predictors of class membership (e.g., demographics, digital literacy) will be explored.

**Estimating willingness to pay and time investment**

Marginal willingness to pay (MWTP) for improvements in attribute levels will be derived by dividing the coefficient of interest by the negative coefficient of the cost variable:

$${MWTP}_{k}=-ß_{k}/ß_{cost}$$

Similarly, marginal willingness to invest time (MTV) will be estimated by:

$${MTV}_{k}=-ß_{k}/ß_{time}$$

These metrics allow for direct economic and behavioral interpretation of stakeholder preferences.

**Software and reproducibility**

The analysis will be conducted using the following software:

- Ngene for experimental design generation,
- Stata 17 for conditional logit and latent class models,
- R (Apollo, support. BWS packages) for mixed logit and BWS estimation.
- Syntax files and codebooks will be made available via an open repository (e.g., OSF) to support transparency and replication.

**Sensitivity and robustness checks**

Robustness of results will be assessed through:

- Re-estimation excluding dominance-task failures,
- Adjusting for attribute non-attendance (ANA),
- Testing alternative coding schemes for attributes,
- Varying parameter distributions in mixed logit models,
- Comparing models with and without outliers or extreme WTP values.

**Fraud detection and response validation**
To detect fraudulent or non-human responses, the following behavioral indicators will be analyzed post hoc:

- Survey completion time, benchmarked against expected reading and response durations.
- Time of participation, with attention to atypical nighttime completions.
- Response patterns, including repetitive or invariant choices across DCE or BWS tasks.
- Open-ended responses, screened for coherence and semantic plausibility.
- Hidden bot-detection item, not visible to human participants but accessible to automated scripts.

These checks ensure the reliability and generalizability of the estimated preferences.
